# Phytofabrication of Silver Nanoparticles (AgNPs) with Pharmaceutical Capabilities Using *Otostegia persica* (Burm.) Boiss. Leaf Extract

**DOI:** 10.3390/nano11041045

**Published:** 2021-04-19

**Authors:** Majid Sharifi-Rad, Pawel Pohl, Francesco Epifano

**Affiliations:** 1 Department of Range and Watershed Management, Faculty of Water and Soil, University of Zabol, Zabol 98613-35856, Iran; 2 Department of Analytical Chemistry and Chemical Metallurgy, Faculty of Chemistry, University of Science and Technology, Wyspianskiego 27, 50-370 Wroclaw, Poland; pawel.pohl@pwr.edu.pl; 3 Dipartimento di Farmacia, Università “G. d’Annunzio” Chieti-Pescara, Via dei Vestini 31, 66100 Chieti Scalo (CH), Italy; fepifano@unich.it

**Keywords:** phytofabrication, silver nanoparticle, medical application, *Otostegia persica*

## Abstract

In the last years, the plant-mediated synthesis of nanoparticles has been extensively researched as an affordable and eco-friendly method. The current study confirms for the first time the capability of the *Otostegia persica* (Burm.) Boiss. leaf extract for the synthesis of silver nanoparticles (AgNPs). The phytofabricated AgNPs were characterized by ultraviolet–visible spectroscopy (UV-Vis), Fourier-transform infrared spectroscopy (FTIR), X-ray diffraction (XRD), transmission electron microscopy (TEM), and zeta potential analysis. Moreover, the total phenolic and flavonoids contents, and the antioxidant, antibacterial, antifungal, and anti-inflammatory properties of the phytofabricated AgNPs and the *O. persica* leaf extract were assessed. The results showed that the produced AgNPs were crystalline in nature and spherical in shape with an average size of 36.5 ± 2.0 nm, and indicated a localized surface plasmon resonance (LSPR) peak at around 420 nm. The zeta potential value of −25.2 mV pointed that the AgNPs were stable. The phytofabricated AgNPs had lower total phenolic and flavonoids contents than those for the *O. persica* leaf extract. The abovementioned AgNPs showed a higher antioxidant activity as compared with the *O. persica* leaf extract. They also exhibited significant antibacterial activity against both Gram-positive (*Staphylococcus aureus*, *Bacillus subtilis*, and *Streptococcus pyogenes*) and Gram-negative (*Escherichia coli*, *Pseudomonas aeruginosa*, and *Salmonella typhi*) bacteria. In addition, appropriate antifungal effects with the minimum inhibitory concentration (MIC) values of 18.75, 37.5, and 75 µg mL^−1^ against *Candida krusei*, *Candida glabrata*, and *Candida albicans*, respectively, were noted for this new bionanomaterial. Finally, the phytofabricated AgNPs showed dose-dependent anti-inflammatory activity in the human red blood cell (RBC) membrane stabilization test, being higher than that for the *O. persica* leaf extract. The resulting phytofabricated AgNPs could be used as a promising antioxidant, antibacterial, antifungal, and anti-inflammatory agent in the treatments of many medical complications.

## 1. Introduction

Nanobiotechnology as an interdisciplinary field is rapidly growing and finds useful applications in physics, chemistry, biology, and biomedicine [1,2]. In particular, various plants and microorganisms have received great attention as helpful resources for the synthesis of nanomaterials [3]. Nanoparticles produced using bacteria-, fungi-, and plants-derived media are more attractive than those obtained with different chemical agents because they are nontoxic, ecofriendly, and safe, especially when considering their potential use in food products and medicines [4]. Although several pathways were developed for the biosynthesis of metal nanoparticles from corresponding metal salts precursors, the most frugal option is to use plant extracts, which is inexpensive, simple, and fast because it has a one-step character and gives products practically ready to use [3,5]. In the latter case, plant extracts act both as reducing agents and as stabilizers of resultant nanoparticles. Nevertheless, the plant extracts’ synthesis ability is differentiated due to certain sources differing, owing to the concentration and composition of specific organic compounds extracted from the plant material [6].

Metal nanoparticles are extensively studied because of their physiochemical properties and biological activity, and hence have potential applications in pharmaceuticals, food coatings, and packaging materials [7]. Among different metallic nanoparticles, silver nanoparticles (AgNPs) are highlighted to be particularly desirable owing to their specific catalytic properties, appropriate conductivity, and a wide range of biological activities, including antioxidant, antimicrobial, anti-inflammatory, and antifungal effects [8,9]. 

*Otostegia persica* (Burm.) Boiss. belongs to the Labiateae family and is a medicinal and endemic plant in Iran. Phytochemical studies of *O. persica* showed that its aerial parts are rich in flavonoids and phenolic compounds such as quercetin, morin, isovitexin, and kaempferol [10]. *O. persica* is traditionally used for treating rheumatism, diabetes, cardiac distresses, hyperlipidemia, gastric discomforts, hypertension, colds, headaches, and addiction treatments [11]. Various biological activities of this medicinal plant were reported, including antimicrobial [12], antioxidant [10], anti-diabetic [13] and anti-inflammatory [14] properties and effects.

Due to the richness of various types of organic compounds, i.e., essential oils (pinene, verbenol, geraniol, eugenol, ceryl alcohol, and hentriacontane), phenolics (morin, quercetin, kaempferol, isovitexin, cinnamic acid, caffeic acid, hydroxy benzoic acid, sitosterol, and sitosteryl acetate), and terpenoids (amyrin, campesterol, and stigmasterol) [15,16], it could be expected that aqueous and alcoholic extracts of this plant would exhibit desired reducing properties toward the Ag(I) ions. In addition, these compounds could efficiently act as capping agents for synthesized AgNPs, providing their higher stabilization and biocompatibility, in addition to beneficial biological activity toward a variety of pathogens. Therefore, the aim of the current study was to evaluate the ability of the ethanolic *O. persica* leaf extract to simply (one-pot) and rapidly (one-step) phytofabricate the AgNPs. The abovementioned AgNPs were synthesized by the *O. persica* leaf extract for the first time and characterized using UV-Vis spectroscopy, Fourier-transform infrared (FTIR) spectroscopy, X-ray diffraction (XRD), transmission electron microscopy (TEM), and zeta potential analysis. Their antioxidant, antibacterial, antifungal, and anti-inflammatory properties were also investigated. 

## 2. Materials and Methods

### 2.1. Plant Material 

Samples of *O. persica* leaves were obtained in May 2020 from Saravan rangelands, Sistan and Baluchistan Province, Iran. The plant material was botanically authenticated at the Department of Rangeland and Watershed Management, University of Zabol, Zabol, Iran, where a voucher specimen (No. 9824) was deposited. The collected samples were washed, shade-dried, and ground to a fine powder by a domestic grinder (Vidas, Tehran, Iran). The ethanolic plant extract was prepared from the abovementioned leaf powder by treating its 5 g with 100 mL of ethanol (98%) for 24 h using a shaker (IKA-Werke GmbH & Co, Staufen, Germany) at a room temperature. The resulting mixture was filtered by a Whatman No. 1 filter paper. The filtrate was kept at a refrigerator temperature (4 ± 1 °C) and utilized for the synthesis of AgNPs as received. In addition, the obtained ethanolic plant extract was evaporated to dryness by using a rotary evaporator under a decreased pressure at 20 to 30 °C and kept at 4 ± 1 °C in dark for further studies.

### 2.2. Phytofabrication of AgNPs

The AgNPs were synthesized as described previously [2,3]. In brief, the ethanolic *O. persica* leaf extract was mixed with a 1 mmol L^−1^ aqueous solution of silver nitrate (AgNO_3_) (Merck, Darmstadt, Germany) at a 1:9 ratio. This reaction mixture was placed into a shaker with a continuous rotation at a room temperature for 150 min. The resulting suspension was centrifuged at 15,000 rpm for 15 min. The obtained pellets were washed twice with deionized water to remove all unconverted Ag(I) ions and a plant extract residue, and then air-dried at a room temperature.

### 2.3. Characterization of the AgNPs

UV–visible absorption spectroscopy was used to identify the presence of phytofabricated AgNPs in the resulting reaction mixtures. UV-Vis absorption spectra of the AgNPs synthesized at various time intervals were acquired in the range from 300 to 800 nm using a UV-1800 Shimadzu spectrophotometer (Shimadzu Corporation, Kyoto, Japan). Distilled water was considered as a blank and applied to zero the spectrophotometer.

For FTIR spectroscopy, a Nicolet 800 FTIR spectrometer (Nicolet, Madison, WI, USA) was used. In this case, the biomolecules present in the ethanolic *O. persica* leaf extract, involved in the reduction of Ag(I) ions, were identified in the frequency range of 500 to 4000 cm^−1^ according to the KBr pellet method as described earlier [2,3]. The dried *O. persica* leaf extract and the dried (and early separated and cleaned) AgNPs were applied as the samples.

X-ray diffraction (XRD) patterns were acquired with a Siemens X-ray diffractometer, model D5000 (Munich, Germany) with a 0.5°/min dwell time. Respective diffractograms of the dried (and early separated and cleaned) AgNPs were acquired in the 2θ range from 20° to 80° by using a Cu–Kα radiation (λ = 1.54 Å) lamp. The Debye–Scherrer’s formula was applied to calculate the average crystallite size of the phytofabricated AgNPs as described previously [17].

The investigation of the shape and size distribution of the AgNPs was done by a Philips GM-30 TEM instrument (Hillsboro, OR, USA) operated at an accelerating voltage of 120 kV. At first, the initially separated and cleaned phytofabricated AgNPs were dispersed in water and sonicated for 5 min. A drop of the resulting suspension was placed on a carbon-coated copper grid, and the solvent was evaporated under an IR lamp for 15 min. For the determination of the particles size distribution based on the TEM images of the AgNPs, Digimizer software (Version 5.4.3, MedCalc Software, Ostend, Belgium) was used. 

The zeta potential of the AgNPs was measured using a Zetasizer Nano ZS (Nano-ZS; Malvern Instruments Ltd., Worcestershire, UK) at 25 °C. The measurement data were analyzed by Zetasizer software. 

### 2.4. Phytochemical Screening

#### 2.4.1. Total Phenolics Content (TPC) Determination

The total concentration of phenolics (TPC) present in the ethanolic *O. persica* leaf extract used for the phytofabrication of the AgNPs and those directly associated with the synthesized AgNPs was determined by the Folin–Ciocalteu method with a slight modification [18]. In brief, the *O. persica* leaf extract and the initially separated and cleaned AgNPs were separately dissolved in distilled water to achieve the final concentrations of 50, 100, 150, 200, 250, and 300 μg mL^−1^. One milliliter of both the *O. persica* leaf extract solution and the AgNPs suspension at different concentrations were mixed with 5 mL of the Folin–Ciocalteu reagent (10%). Afterward, 4 mL of a Na_2_CO_3_ solution (2%) was added, and the resulting mixtures were incubated in dark at a room temperature for 1 h. The absorbance of the resulting reaction mixtures was measured at 760 nm with the aid of a Shimadzu UV-Vis spectrophotometer, model UV-1800, against the control, being a mixture of both reagents but without the extract and the AgNPs added. Gallic acid was used to prepare standard solutions for the calibration and quantification of the TPC in the *O. persica* leaf extract and the phytofabricated AgNPs. The results were expressed as microgram (μg) of the GAE (gallic acid equivalent).

#### 2.4.2. Total Flavonoid Content (TFC) Measurement

The total content of flavonoids (TFC) present in the *O. persica* leaf extract and those directly associated with the phytofabricated AgNPs was measured by the aluminum chloride (AlCl_3_) colorimetric method [18]. Accordingly, 0.5 mL of the *O. persica* leaf extract solutions and the suspensions of the initially separated and cleaned AgNPs, both at concentrations of 50, 100, 150, 200, 250, and 300 μg mL^−1^, were separately blended with 1.5 mL of methanol, 0.1 mL of a 10% AlCl_3_ solution, 0.1 mL of a potassium acetate solution (1 mol L^−1^), and 2.8 mL of distilled water. The resulting reaction mixtures were kept at a room temperature for 30 min, and then their absorbance was measured at 415 nm using a Shimadzu UV-Vis spectrophotometer, model UV-1800. Quercetin was considered as the standard for the calibration and the quantification of the TFC. The results were expressed as microgram (μg) of the QE (quercetin equivalent).

### 2.5. Antioxidant Potential

#### 2.5.1. 2,2-diphenyl-1-picrylhydrazyl (DPPH) Radical Scavenging Activity Assay

The DPPH radical scavenging activity of the *O. persica* leaf extract and the synthesized AgNPs was expressed as the inhibition percentage in the DPPH radical and evaluated according to the method described by Sharifi-Rad et al. [19]. In summary, 0.4 mL of the *O. persica* leaf extract solutions and the suspensions of the phytofabricated AgNPs, both at concentrations of 50, 100, 150, 200, 250, and 300 μg mL^−1^, were mixed with 3 mL of a 0.1 mmol L^−1^ DPPH radical working solution (as a source of free radicals). Next, the resulting reaction mixtures were kept at a room temperature for 30 min, and afterwards, the absorbance of the prepared samples solutions was recorded at 517 nm using a Shimadzu UV-Vis spectrophotometer, model UV-1800. The solutions of butylated hydroxyanisole (BHA) at the same concentrations (50, 100, 150, 200, 250, and 300 μg mL^−1^) were used for the comparison. Just the DPPH radical working solution was used as the control. The DPPH radical scavenging activity (%) was determined using the following equation:

Inhibition in DPPH (%) = [(Absorbance_(Control)_ − Absorbance_(sample)_)/Absorbance_(Control)_] × 100. (1)

#### 2.5.2. 2,2′-azino-bis(3-ethylbenzothiazoline-6-sulfonic acid) (ABTS) Radical Scavenging Activity Assay

The ABTS radical scavenging activity of the *O. persica* leaf extract and the synthesized AgNPs was expressed as the percentage inhibition in the ABTS radical and evaluated according to the method described by Min-Jung et al. [20] with a slight modification. To prepare an ABTS radical working solution, equal volumes of a 7.0 mmol L^–1^ ABTS radical solution and a 2.4 mmol L^−1^ potassium persulfate (K_2_S_2_O_8_) solution were mixed, and the resultant mixture was kept in dark at a room temperature for 16 h. The obtained solution was diluted in ethanol to achieve the ABTS radical working solution for which the absorbance of 0.7 was measurable at 734 nm. *O. persica* leaf extract solution (100 μL) and the suspension of the phytofabricated AgNPs (100 μL), both at various concentrations, i.e., 50, 100, 150, 200, 250, and 300 μg mL^−1^, were separately added to 3 mL of the ABTS radical working solution. The absorbance of the resulting samples solutions was measured at 734 nm after 30 min, using a Shimadzu UV-Vis spectrophotometer, model UV-1800, against a respective control, i.e., the ABTS radical working solution. The solutions of BHA at the same concentrations as the *O. persica* leaf extract or the phytofabricated AgNPs were used for the comparison. All solutions were daily prepared and immediately applied. The ABTS radical scavenging activity (%) was determined in the same way as the inhibition in DPPH.

### 2.6. Antibacterial Assays

The antibacterial activity of the *O. persica* leaf extract and the synthesized AgNPs was evaluated using Gram-positive (*Staphylococcus aureus* (ATCC 25923), *Bacillus subtilis* (ATCC 6633), and *Streptococcus pyogenes* (ATCC 12344)) and Gram-negative (*Escherichia coli* (ATCC 25922), *Pseudomonas aeruginosa* (ATCC 9027), and *Salmonella typhi* (ATCC 19430)) bacteria. The bacteria strains were prepared from Iranian Research Organization for Science and Technology (IROST). 

#### 2.6.1. Disc Diffusion Method

The disc diffusion assay was applied to screen the antimicrobial activity of the *O. persica* leaf extract and the synthesized AgNPs as described by the Sharifi-Rad et al. [21]. The sterilized paper discs (diameter of 6 mm) were impregnated with 20 µL of the *O. persica* leaf extract solutions and the suspensions of the phytofabricated AgNPs at various concentrations (50, 100, 150, 200, 250, and 300 μg mL^−1^), and then air-dried. The blank disks were impregnated with solvents (water) and used as negative controls. To inoculate the Müller-Hinton agar (MHA) plates, 0.5 McFarland bacteria suspensions (consisting of 1.5 × 10^8^ CFU mL^−1^ of studied bacteria strains) were applied. The paper disks were placed on the surface of these MHA plates. Gentamicin (10 µg/disk) served as the positive control. All the inoculated plates by bacteria were incubated at 37 °C for 24 h. After the incubation, the antibacterial activity was investigated through measuring the clear zones of inhibition to the nearest millimeter (mm).

#### 2.6.2. Measurement of the Minimum Inhibitory Concentration (MIC)

The minimum inhibitory concentration (MIC) for the *O. persica* leaf extract and the synthesized AgNPs was determined according to the guidelines given by the Clinical and Laboratory Standards Institute [22]. The concentrations of the *O. persica* leaf extract solutions and the suspensions of the phytofabricated AgNPs applied for the determination of the MIC were varied from 300 to 2.35 µg mL^−1^. Different concentrations of the *O. persica* leaf extract (50 µL) and of the Müller-Hinton broth (50 µL) were poured into each well of a polystyrene 96-well plate. Using another polystyrene 96-well plate, 50 µL of the different concentrations of the phytofabricated AgNPs and 50 µL of the Müller-Hinton broth were dumped into the wells. Eventually, 50 µL of a 0.5 McFarland bacteria suspension (consisting 1.5 × 10^8^ CFU/mL of bacteria) was added into the wells. The inoculated plates were incubated at 37 °C for 24 h. The MIC values explained the lowest concentrations of the *O. persica* leaf extract or the synthesized AgNPs that suppressed the visible growth of each tested bacteria strain.

#### 2.6.3. Measurement of the Minimum Bactericidal Concentration (MBC)

The determination of the minimal bactericidal concentration (MBC) was carried out according to the method described by the Clinical and Laboratory Standards Institute [22] too. A liquid portion (50 μL) from each well of the broth micro-dilution method that indicated no visible bacterial growth was taken and spread evenly over the MHA plates, and then incubated at 37 °C for 24 h. The lowest concentrations of the *O. persica* leaf extract or the phytofabricated AgNPs that exhibited no bacterial growth were considered as the MBC.

### 2.7. Antifungal Potential

The antifungal activity of the *O. persica* leaf extract and the synthesized AgNPs was assayed against the standard *Candida* strains, including *C. krusei* (ATCC 6258), *C. albicans* (ATCC 14053), and *C. glabrata* (ATCC 90030). All strains were obtained from the Iranian Research Organization for Science and Technology (IROST) and were incubated overnight on a Sabouraud dextrose agar (Merck KGaA, Darmstadt, Germany) consisting of chloramphenicol (5%).

#### Antifungal Susceptibility Test

The antifungal susceptibility against the studied strains was determined using the broth micro-dilution method as presented by Quan et al. [23]. The concentrations of the *O. persica* leaf extract solutions and the suspensions of the phytofabricated AgNPs considered for the minimum inhibitory concentrations (MICs) were varied from 300 to 2.35 µg mL^−1^, while the initial fungi concentration suspended in the RPMI 1640 medium (Sigma, St. Louis, MO, USA) was 2.5 × 10^3^ cells mL^−1^. The wells containing the fungi inoculum without the *O. persica* leaf extract or the phytofabricated AgNPs were intended as negative controls. The wells containing the fungi inoculum and added fluconazole (300 to 2.35 µg mL^−1^) were considered as positive controls. The polystyrene 96-well plates were incubated at 35 °C for 48 h, then the MIC values were determined visually and also by measuring the optical density of each well using a microplate reader (BioTek, Winooski, VT, USA) at 405 nm. To evaluate the minimum fungicidal concentrations (MFCs), 50 μL of the culture from each well of the micro-dilution method that exhibited no visible fungal growth was sub-cultured on the Sabouraud dextrose agar and incubated at 35 °C for 24 h. The lowest concentrations of the *O. persica* leaf extract or the synthesized AgNPs that showed no fungal growth were intended as the MFCs.

### 2.8. Anti-Inflammatory Assay

#### Human Red Blood Cell Stabilization Method

The human red blood cell (RBC) membrane stabilization assay was applied to investigate the anti-inflammatory activity of the *O. persica* leaf extract and the phytofabricated AgNPs as described by Vane and Botting [24]. The blood samples were obtained from ten healthy volunteers. The samples were blended with an equal volume of a sterilized Alsever’s solution (containing 0.5% citric acid, 0.8% sodium citrate, 0.42% sodium chloride, and 2% dextrose). To separate the packed cells, the preserved blood samples were centrifuged at 4000 rpm for 15 min. The obtained packed cells were washed with 0.85% isosaline (pH 7.2), and a cell suspension (10% *v/v*) was prepared in isosaline. This resulting human RBC suspension was applied for the determination of the anti-inflammatory activity. Accordingly, 1 mL amounts of the *O. persica* leaf extract solutions, the suspensions of the phytofabricated AgNPs, and the diclofenac sodium solutions (as a standard drug) at various concentrations (50, 100, 150, 200, 250, and 300 μg mL^−1^) were separately blended with 2 mL of 0.36% hypo saline, 1 mL of a 0.15 mol L^−1^ phosphate buffer (pH 7.4), and 0.5 mL of the human RBC suspension. Distilled water (2 mL) was considered as the control instead of the hypo saline. The resulting mixtures were incubated at 37 °C for 30 min and then centrifuged at 4000 rpm for 15 min. The supernatants were evacuated, and their hemoglobin value was spectrophotometrically measured at 560 nm. The protection (%) or the human RBC membrane stabilization (%) was determined using the following equation:

Protection (%) = 100 − (OD_test_/OD_control_) × 100. (2)

### 2.9. Statistical Analysis

The statistical analysis was done by the SPSS software (Version 11.5, SPSS Inc., Chicago, IL, USA). The analysis of variance (ANOVA) followed by the Duncan’s multiple range test was used with 95% significance level (α = 0.05). All data were expressed as mean ± SD values. All experiments were carried out in triplicate. 

## 3. Results and Discussion

### 3.1. Visual Confirmation of the Phytofabrication of AgNPs

**Figure 1:**
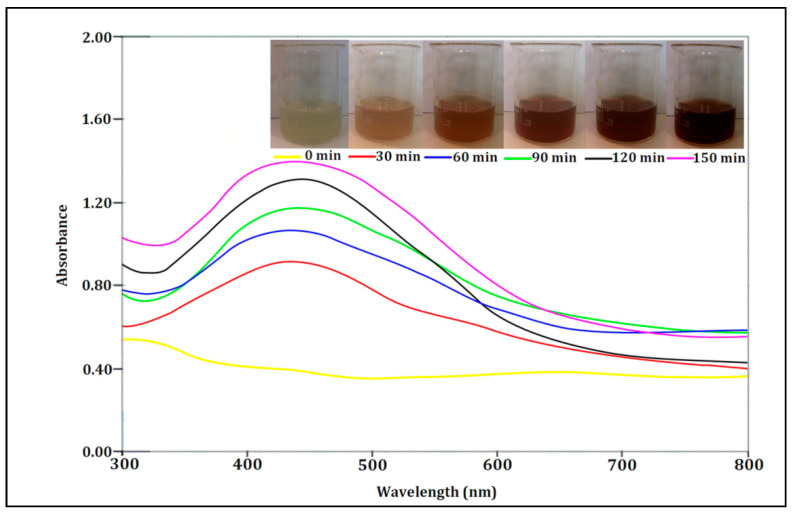
The UV-Vis absorption spectra of the AgNPs phytofabricated by using the *O. persica* leaf extract and the color change of the reaction mixture due to the formation of the AgNPs at different reaction times.

The phytofabrication process of the AgNPs was performed at a room temperature and under low-light conditions to reduce the photo-activation effect of the Ag(I) ions. The color change of the colorless AgNO_3_ solution to dark brown after the addition of the *O. persica* leaf extract proved that the phytofabrication of the AgNPs occurred [3,9] (Figure 1). This confirmed that the applied plant extract contained the reducing agents capable of the reduction of the Ag(I) ions into the AgNPs and their stabilization in the solution.

### 3.2. The Phytofabricated AgNPs Characterization 

#### 3.2.1. UV-Vis Spectroscopy

UV-Vis absorption spectroscopy was initially used to investigate the optical response of the phytofabricated AgNPs because of their intense surface plasmon resonance (SPR) in the Vis region, confirming their efficient formation [25]. The reduction rate of the Ag(I) ions into the AgNPs by the organic compounds present in the *O. persica* leaf extract was monitored versus time (30, 60, 90, 120, and 150 min) (Figure 1). It was found that the maximum absorption peak in the UV-Vis absorption spectra of the reaction mixtures, attributed to the SPR band of the phytofabricated AgNPs, was recorded at 420 nm, which indicated the production of the spherical nanoparticles. Similarly to the results of this study, many previous studies pointed out that the SPR peak located between 410 and 460 nm was related to the formation and presence of the spherical and/or near-spherical AgNPs [26,27]. In addition, it was also found that the intensity of the SPR band of the phytofabricated AgNPs, related to the reaction efficiency, gradually increased with time. Hence, the highest phytofabrication efficiency of the AgNPs was likely achieved 150 min after the mixing of the precursor solution (AgNO_3_) and the plant extract studied here. UV-Vis spectra of the mixtures were also obtained at times higher than 150 min. These spectra were overlapped with the spectra obtained at 150 min that indicated that the reaction was ended.

#### 3.2.2. FTIR Spectroscopy

**Figure 2:**
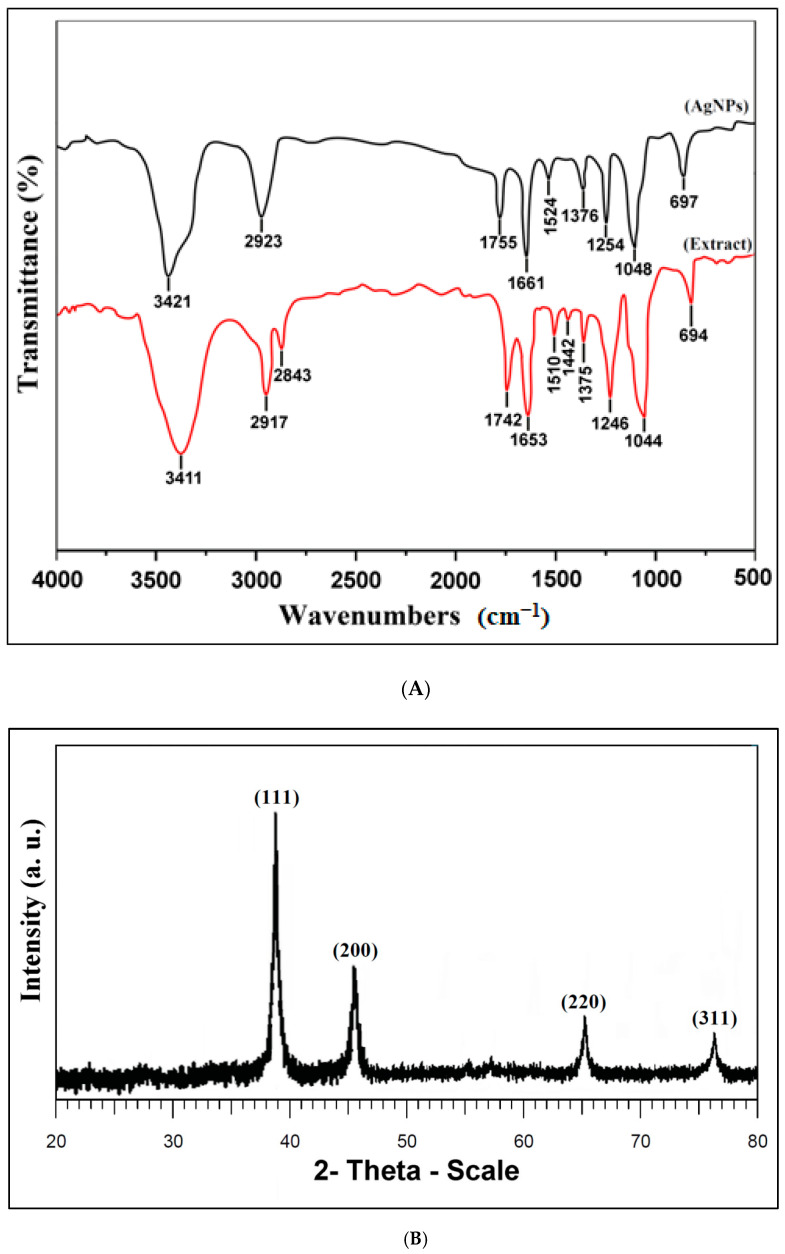
(**A**) The Fourier-transform infrared (FTIR) spectrum of the *O. persica* leaf extract (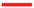) and the phytofabricated AgNPs (separated from the reaction mixture and cleaned with water) (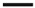); (**B**) The X -ray diffraction (XRD) pattern of the AgNPs phytofabricated by using the *O. persica* leaf extract.

The FTIR spectroscopy analysis was performed to identify the possible organic biomolecules of the *O. persica* leaf extract that were responsible for the phytofabrication of the spherical AgNPs (Figure 2A). In the FTIR spectrum in the range from 500 to 4000 cm^−1^ of such phytofabricated AgNPs, the following absorption peaks were observed: 3421, 2923, 1755, 1661, 1524, 1376, 1254, 1048, and 697 cm^−1^. These absorption peaks were attributed to the O–H stretching vibrations (3421 cm^−1^) in alcohols, phenols, and flavonoids compounds [28], the C–H stretching vibrations (2923 cm^−1^) of aromatic compounds [27], the C=O stretching vibrations (1755 cm^−1^) for carbonyl compounds [29], the N–H stretching vibrations (1661 cm^−1^) of amide (I) in proteins [30], the C=C stretching vibrations (1524 cm^−1^) of aromatic compounds [31], the C–H stretching vibrations (1376 cm^−1^) in methylene moieties [32], the C–N stretching vibrations (1254 cm^−1^) of aliphatic amines [33], the C–O stretching vibrations (1048 cm^−1^) in alcohols and phenols [34], and finally, the C–H bonding vibrations (697 cm^−1^) of aromatic compounds [35]. In the FTIR spectrum of the *O. persica* leaf extract, the corresponding absorption peaks were observed at 3417, 2917, 2843, 1742, 1653, 1510, 1442, 1375, 1246, 1044, and 694 cm^−1^, and as before related to the appropriate vibrations of the O–H, C–H, C–H, C=O, N–H, C=C, –OH, C–H, C–N, C–O, and C–H groups, respectively. In general, all the identified absorption peaks were shifted in the AgNPs FTIR spectrum when compared with their observed location in the *O. persica* leaf extract spectrum. This confirmed that various functional groups and moieties of the organic compounds present in the *O. persica* leaf extract contributed to the reduction of the Ag(I) ions (leading to the AgNPs synthesis) and the capping of the resultant nanostructures (providing their stability in time and functionality, likely related to their biocompatibility and biological activity) [2].

#### 3.2.3. X-ray Diffraction (XRD)

The crystalline structure of the AgNPs phytofabricated by the *O. persica* leaf extract was evaluated through the XRD analysis (Figure 2B). The 2θ peaks identified in the diffractogram were at 38.4°, 45.7°, 65.2°, and 76.3°; they were attributed to the (111), (200), (220), and (311) Miller indices, respectively. As such, the observed AgNPs diffraction peaks and respectively assigned Miller indices indicated the face-centered cube (FCC) crystalline structure of the phytofabricated Ag material (according to the database in the Joint Committee on Powder Diffraction Standards (JCPDS) library, No. 04-0783). The observed sharpening of the diffraction peaks clearly confirmed that the produced Ag crystalline material was in the nanoparticle regime [36]. Using the Debye–Scherrer formula, the average crystallite size of the abovementioned AgNPs was calculated to be 37.2 nm.

#### 3.2.4. Transmission Electron Microscopy (TEM)

**Figure 3:**
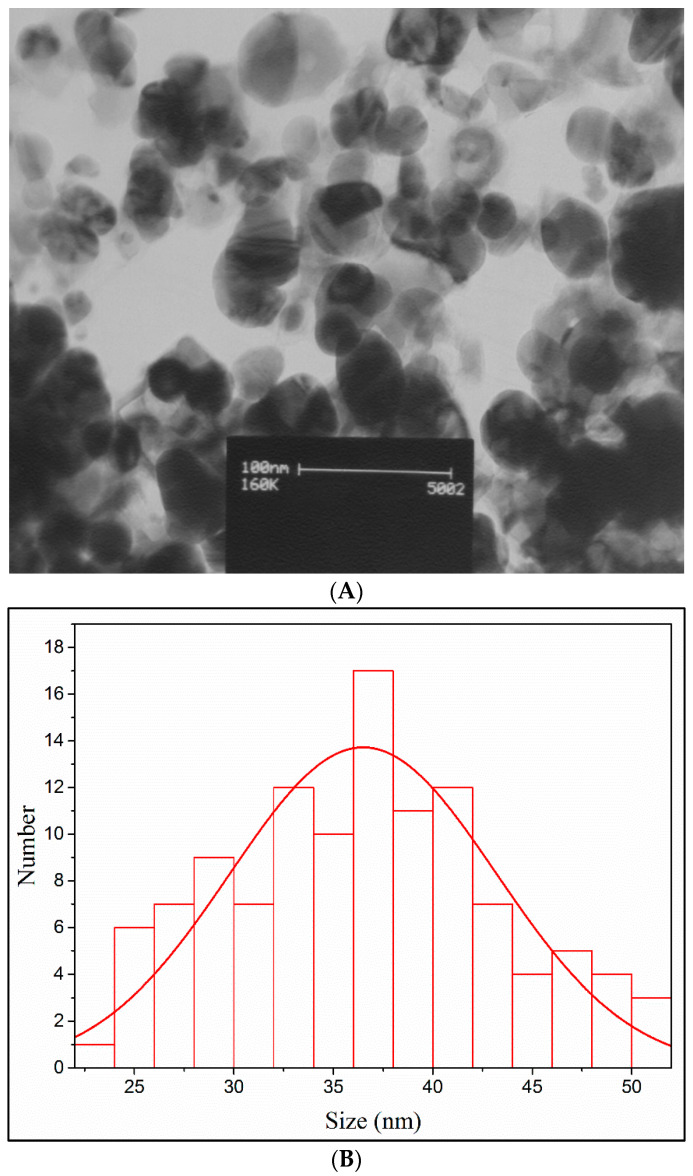
(**A**) The TEM image, and (**B**) the particles size distribution of the AgNPs phytofabricated by using the *O. persica* leaf extract.

TEM was applied to quantitatively measure the size, morphology, and the size distribution of the separated and cleaned AgNPs [37]. The TEM analysis (Figure 3A,B) confirmed that the AgNPs produced by the *O. persica* leaf extract had a spherical structure and a particle size distribution that varied from 23.4 to 53.2 nm. The average size of the phytofabricated AgNPs was 36.5 ± 2.0 nm, which corresponded to the average size assessed based on the XRD analysis.

#### 3.2.5. Zeta Potential

**Figure 4:**
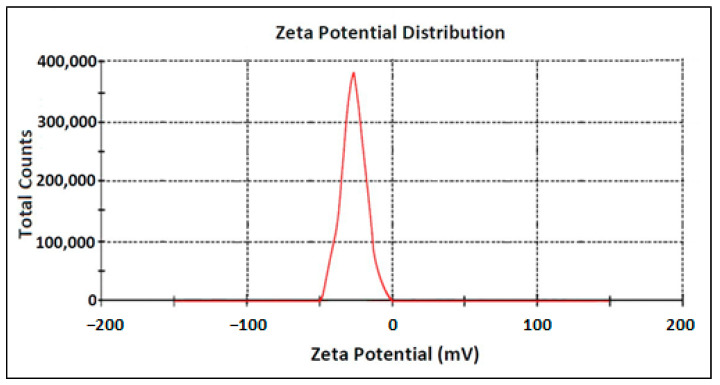
The zeta potential of the AgNPs phytofabricated by using the *O. persica* leaf extract.

Since the zeta potential value can provide evidence about the capping agent’s efficiency for the stabilization of the nanoparticles through creating an intensive negative charge [38], the zeta potential analysis of the phytofabricated AgNPs obtained in the present work was carried out. It was established that the phytofabricated AgNPs had a negative zeta potential of nearly −25.2 mV (Figure 4). This negative surface charge of the produced AgNPs was likely attributed to the adsorption of the bioactive compounds present in the *O. persica* leaf extract onto their surface and confirmed a relatively high biocompatibility of the nano material produced [39].

### 3.3. Phytochemical Analysis

#### Total Phenolic and Flavonoid Contents

**Figure 5:**
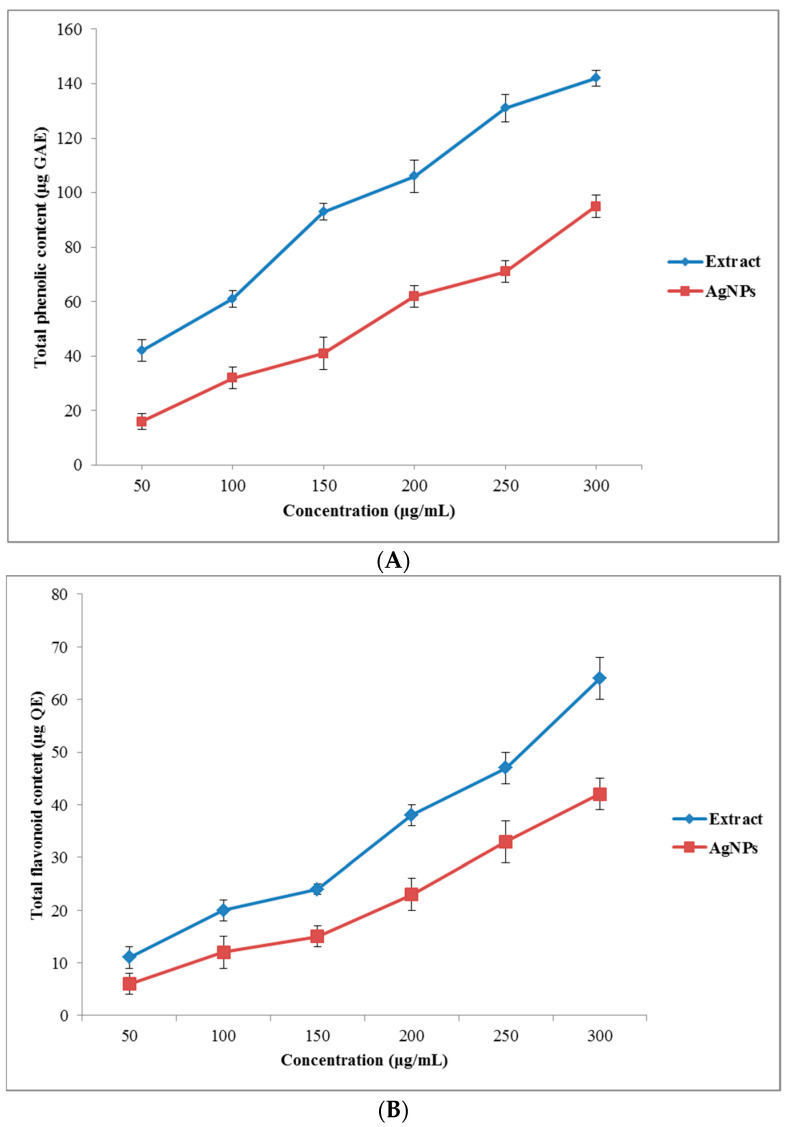
(**A**) The total phenolics and (**B**) the total flavonoids content of the *O. persica* leaf extract and the phytofabricated AgNPs.

Secondary metabolites of plant extracts were reported to play an important role in the phytofabrication of metallic NPs due to their ability to reduce the metal ions into respective nanostructures of different size and morphology, and support the subsequent stability of the produced metallic NPs [40]. The TPC and the TFP of the *O. persica* leaf extract and the produced AgNPs were increased in a dose-dependent manner (Figure 5A,B). The TPC of the *O. persica* leaf extract and the phytofabricated AgNPs ranged from 42 ± 4 to 142 ± 4 μg of GAE and from 16 ± 3 to 95 ± 4 μg of GAE, respectively. Their TFC values varied from 11 ± 2 to 64 ± 4 μg of QE (the plant extract) and from 6 ± 2 to 42 ± 3 μg of QE (the AgNPs). These results confirmed that the produced AgNPs were capped by the phenolic and flavonoid compounds, which were involved in the reduction of the Ag(I) ions and the stabilization of the shape and size of the produced nanomaterial, and also determined their functionality and biological activity [41].

### 3.4. Antioxidant Activity

#### 3.4.1. DPPH Radical Scavenging Activity

**Figure 6:**
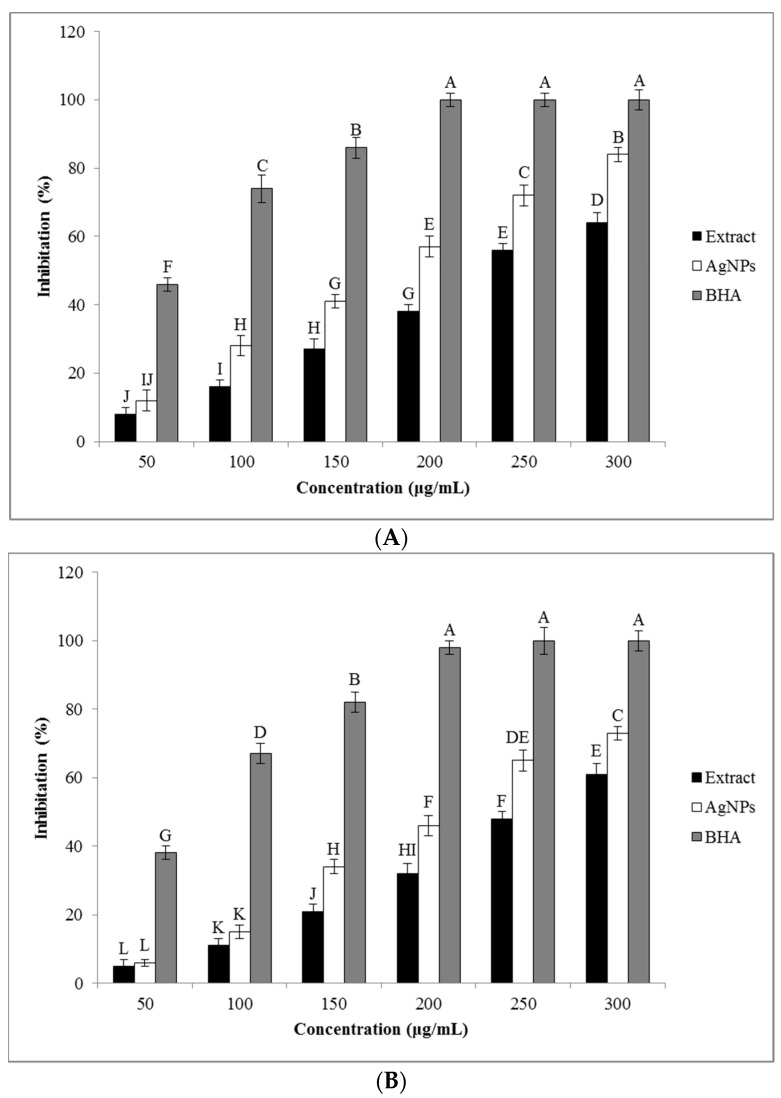
(**A**) The 2,2-diphenyl-1-picrylhydrazyl (DPPH) radical scavenging activity and (**B**) the 2,2’-azino-bis(3-ethylbenzothiazoline-6-sulfonic acid) (ABTS) radical scavenging activity of the *O. persica* leaf extract and the phytofabricated AgNPs. Various letters denote significant differences among the treatments based on the Duncan’s test (*p* < 0.05).

The DPPH radical scavenging activity, known as the ability of antioxidants to donate H atoms to radicals, responsible for their stabilization and unreactivity [42], was measured both for the *O. persica* leaf extract and for the phytofabricated AgNPs. The results on the DPPH radical-scavenging activity obtained for the analyzed solutions and suspensions containing correspondingly different concentrations of the *O. persica* leaf extract and the phytofabricated AgNPs are shown in Figure 6A. It can be seen that the free radical-scavenging activity of the *O. persica* leaf extract and the phytofabricated AgNPs gradually increased with increasing their concentration. Notably, the phytofabricated AgNPs indicated the highest antioxidant activity of 84% at the highest studied concentration of 300 µg mL^−1^. It was higher as compared with the highest value that was achievable for the *O. persica* leaf extract (64%). This was a clear sign that the activity of the produced AgNPs was not only because of their capping with the phytochemical compounds of the *O. persica* leaf extract, but was also because of the elemental Ag [43].

#### 3.4.2. ABTS Radical Scavenging Activity

The results on the ABTS radical scavenging activity of the *O. persica* leaf extract and the phytofabricated AgNPs are presented in Figure 6B. The phytofabricated AgNPs indicated the maximal ABTS scavenging activity value of 73% at 300 µg mL^−1^, whereas for the same concentration of the *O. persica* leaf extract it was 61%. Since the ABTS method is often applied for measuring the antioxidant properties of the H-atoms-donating and chain-breaking antioxidants in phytomedicine studies [44], the obtained results showed that the potent antioxidant activity of the produced AgNPs was lower than that for a standard drug (BHA). Nevertheless, the stronger antioxidant activity of the produced AgNPs than assessed for the *O. persica* leaf extract might be attributed to a synergistic effect and an active physicochemical interaction between the functional groups of the organic compounds originating from the *O. persica* leaf extract and capping the AgNPs as well as the Ag atoms themselves from this new phytofabricated nanomaterial and a large surface area of this nanomaterial [45].

### 3.5. Antibacterial Activity

#### 3.5.1. Disc Diffusion Method

The antibacterial activity of the *O. persica* leaf extract and the phytofabricated AgNPs was evaluated on a range of pathogenic microorganisms, using the agar disk diffusion assay. The inhibition zone diameters (mm) are shown in Table 1. The results showed that the *O. persica* leaf extract and the phytofabricated AgNPs have a good antibacterial activity against the tested bacteria strains. The phytofabricated AgNPs indicated a higher antibacterial activity than that determined for the *O. persica* leaf extract. In both cases, the antibacterial activity was increased when increasing the concentration of the plant extract and the AgNPs. It was found that the phytofabricated AgNPs exhibited a stronger biocidal activity against the Gram-negative bacteria as compared with the Gram-positive bacteria strains. These findings are consistent with the results of the previous studies, also focusing on the antibacterial activity of the greenly synthesized AgNPs [2]. The antibacterial effect of the produced AgNPs could be explained by considering their small size and an extremely large surface area-to-volume ratio, providing better conditions for the interaction with bacterial cells [46]. Furthermore, the Ag(I) ions could be easily released from such AgNPs and then more effectively penetrate into the bacteria cell membranes and interact with the intercellular biomolecules such as DNA and proteins [47]. As a result, the replication of the bacterial DNA would be efficiently inhibited, leading to the loss of the cell viability and, in the extreme cases, to the cell death [47].

#### 3.5.2. Minimum Inhibitory Concentration (MIC) and Minimum Bactericidal Concentration (MBC)

The results on the MIC and the MBC values determined for the *O. persica* leaf extract and the phytofabricated AgNPs are listed in Table 2. In agreement with the results of the first antibacterial test carried out in the present work, i.e., the disc diffusion method, the results of the micro-dilution method confirmed that the tested bacteria strains were more susceptible to the effect of the phytofabricated AgNPs than to the *O. persica* leaf extract. The MIC values of the produced AgNPs against the studied bacteria strains ranged from 9.4 to 37.5 μg mL^−1^, while for the *O. persica* leaf extract these values varied from 75 to 150 μg mL^−1^. The MBC values assessed for the produced AgNPs and the *O. persica* leaf extract ranged from 18.75 to 75 μg mL^−1^ and from 150 to 300 µg mL^−1^, respectively.

### 3.6. Antifungal Activity

**Figure 7:**
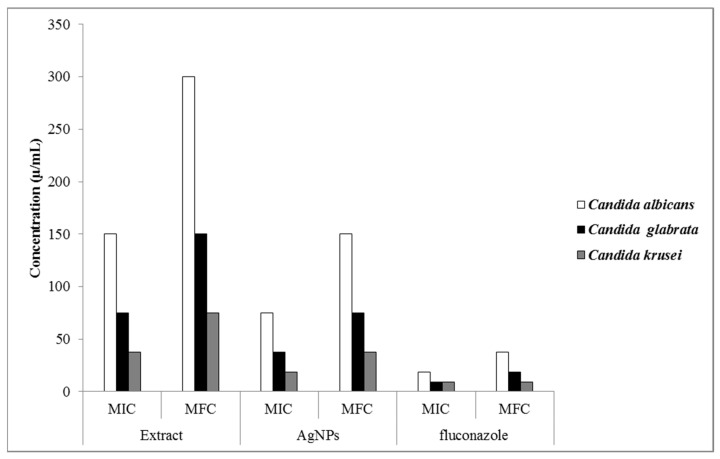
The antifungal activity of the *O. persica* leaf extract and the phytofabricated AgNPs.

As shown in Figure 7, the produced AgNPs indicated a stronger antifungal activity against all studied fungi than that determined for the *O. persica* leaf extract with the MIC values varying from 18.75 to 75 μg mL^−1^. The MFC values of the produced AgNPs and the *O. persica* leaf extract were changed from 37.5 to 150 μg mL^−1^ and from 75 to 300 μg mL^−1^, respectively. The desirable biocidal properties of the AgNPs were likely attributed to their shape, size, and the surface coating [48]. The phytofabricated AgNPs, as proved to be capped by different organic compounds, could cause several concurrent types of metabolic and structural damages in the *Candida* cells, like the disruption of the membrane [49], the depolarization of the membrane [50], and the inhibition of enzymatic functions [51]. This widespread disruption of the cell structure and its function certainly decreased their resistance to the antifungal effect of the phytofabricated AgNPs.

### 3.7. Anti-Inflammatory Activity

#### Human Red Blood Cell Stabilization 

**Figure 8:**
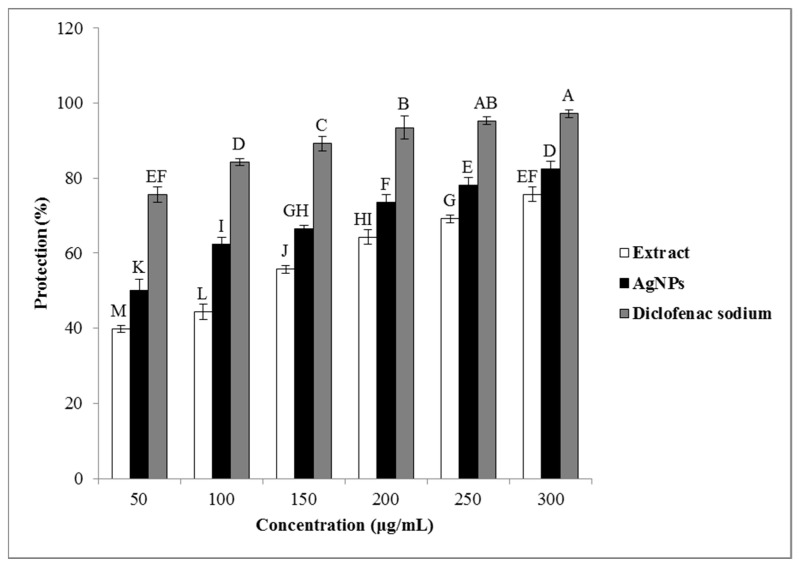
The anti-inflammatory activity of the *O. persica* leaf extract and the phytofabricated AgNPs. Various letters denote significant differences among the treatments based on the Duncan’s test (*p* < 0.05).

The results of the anti-inflammatory activity analysis of the *O. persica* leaf extract and the phytofabricated AgNPs are presented in Figure 8. According to these results, the produced AgNPs exhibited the stronger stabilizing properties on the human RBC membrane as compared with those for the *O. persica* leaf extract. The membrane-stabilizing properties of the phytofabricated AgNPs were established to be concentration-dependent and varied from 50.1% to 82.5%. In the inflammation process, the lysosomal enzymes are released inside the cytosol and damage the nearby tissues, leading to various disorders [52]. Nonsteroidal anti-inflammatory drugs commonly stabilize lysosomal membranes or inactivate the released enzymes [53]. The structure of the human RBC membranes was reported to be similar to that of the lysosomal membranes [54]. Therefore, the lysosomal membrane stability properties can be investigated by the human RBC [55]. When the human RBC is under a hypotonic stress, the hemoglobin release from the RBC is prevented by using the anti-inflammatory agents due to the membrane stabilization [53]. Therefore, the drug stabilization of the human RBC membrane against the hypotonicity-induced hemolysis is used as a beneficial assay to evaluate the anti-inflammatory activity of various compounds [56]. In this study, the phytofabricated AgNPs demonstrated appropriate hemolytic properties on the human RBC membrane and exhibited the good stabilizing properties on the hypotonicity-induced human RBC membrane in addition to their good anti-inflammatory activity.

## 4. Conclusions

The results of the current study indicated that the stable-in-time AgNPs were successfully synthesized at a room temperature by using the *O. persica* leaf extract. The average size of these phytofabricated nanostructures was 36.5 nm, and they were spherical in shape. As confirmed by UV-Vis spectroscopy, FTIR spectroscopy, XRD, TEM, and zeta potential analyses, the utilization of the *O. persica* leaf extract for the phytofabrication of the AgNPs is inexpensive, simple, and fast (one-step and one-pot synthesis), as well as environment friendly (less energy consumption). As a result, the nontoxic and safe AgNPs were produced as required for the food and therapeutic applications. Indeed, the phytofabricated AgNPs displayed good antioxidant, antibacterial, antifungal, and anti-inflammatory activities. Therefore, the *O. persica* leaf extract could be effectively applied in medical healthcare fields for the design of newer drugs based on metallic nanoparticles. On the other hand, the resulting AgNPs could find an important place in biomedical applications, especially antimicrobial applications, offering protection from bacterial and fungal degradation as well as prophylactic and therapeutic effects in the case of bacterial and fungal diseases and infections.

## Figures and Tables

**Table 1:** The inhibition zone of the *O. persica* leaf extract and the phytofabricated AgNPs against Gram-positive and Gram-negative bacteria strains.

		Diameter of the Inhibition Zone (mm)					
	Concentration (µg/mL)	*Staphylococcus* *aureus*	*Bacillus subtilis*	*Streptococcus pyogenes*	*Escherichia* *Coli*	*Pseudomonas aeruginosa*	*Salmonella typhi*
Extract	50	13 ± 0.5 ^Ka^	12 ± 0.4 ^Ib^	10 ± 0.4 ^Gc^	7 ± 0.2 ^Lf^	9 ± 0.2 ^Ld^	8 ± 0.1 ^Le^
	100	15 ± 0.3 ^Ja^	13 ± 0.5 ^Hb^	12 ± 0.1 ^Fc^	9 ± 0.4 ^Kf^	11 ± 0.4 ^Kd^	10 ± 0.2 ^Ke^
	150	17 ± 0.1 ^Ha^	15 ± 0.3 ^Gb^	14 ± 0.2 ^Ec^	10 ± 0.4 ^Jf^	12 ± 0.3 ^Jd^	11 ± 0.3 ^Je^
	200	19 ± 0.2 ^Fa^	17 ± 0.2 ^Fb^	15 ± 0.6 ^Dc^	12 ± 0.5 ^If^	14 ± 0.5 ^Id^	13 ± 0.4 ^Ie^
	250	20 ± 0.3 ^Ea^	18 ± 0.2 ^Eb^	17 ± 0.2 ^Cc^	14 ± 0.2 ^Hf^	16 ± 0.2 ^Hd^	15 ± 0.2 ^He^
	300	22 ± 0.2 ^Ca^	19 ± 0.3 ^Db^	18 ± 0.3 ^Bc^	15 ± 0.3 ^Ge^	18 ± 0.3 ^Gc^	17 ± 0.5 ^Gd^
AgNPs	50	15 ± 0.3 ^Jd^	13 ± 0.2 ^He^	12 ± 0.3 ^Ff^	16 ± 0.2 ^Fc^	19 ± 0.3 ^Fa^	17 ± 0.6 ^Gb^
	100	16 ± 0.1 ^Id^	15 ± 0.3 ^Ge^	14 ± 0.4 ^Ef^	17 ± 0.1 ^Ec^	21 ± 0.5 ^Ea^	19 ± 0.4 ^Eb^
	150	18 ± 0.4 ^Gd^	17 ± 0.5 ^Fe^	15 ± 0.6 ^Df^	19 ± 0.3 ^Dc^	22 ± 0.2 ^Da^	20 ± 0.2 ^Db^
	200	20 ± 0.2 ^Ec^	18 ± 0.4 ^Ed^	17 ± 0.2 ^Ce^	21 ± 0.4 ^Cb^	23 ± 0.1 ^Ca^	21 ± 0.3 ^Cb^
	250	21 ± 0.5 ^Dd^	20 ± 0.2 ^Ce^	18 ± 0.1 ^Bf^	23 ± 0.2 ^Bc^	26 ± 0.1 ^Ba^	24 ± 0.2 ^Bb^
	300	23 ± 0.4 ^Bd^	21 ± 0.2 ^Be^	20 ± 0.1 ^Af^	25 ± 0.6 ^Ac^	28 ± 0.3 ^Aa^	26 ± 0.1 ^Ab^
Gentamicin (10 µg/disk)		25 ± 0.2 ^Aa^	22 ± 0.1 ^Ab^	20 ± 0.5 ^Ac^	16 ± 0.2 ^Ff^	19 ± 0.4 ^Fd^	18 ± 0.2 ^Fe^

Various uppercase letters show significant differences among different concentrations of the extract and the AgNPs, and various lowercase letters show significant differences among different bacteria at the same concentration (*p* < 0.05).

**Table 2:** The minimum inhibitory concentrations (MIC) and the minimum bactericidal concentrations (MBC) of the *O. persica* leaf extract and the phytofabricated AgNPs against Gram-positive and Gram-negative bacteria.

	MIC (µg/mL)		MBC (µg/mL)	
Bacteria strains	Extract	AgNPs	Extract	AgNPs
*Staphylococcus aureus*	75	37.5	150	75
*Bacillus subtilis*	75	37.5	150	75
*Streptococcus pyogenes*	75	37.5	150	75
*Escherichia coli*	150	18.75	300	37.5
*Pseudomonas aeruginosa*	150	9.4	300	18.75
*Salmonella typhi*	150	9.4	300	18.75

## Data Availability

The data presented in this study are available on request from the corresponding author.
